# Outcomes after extracorporeal life support for COVID-19 myocarditis: an analysis of the Extracorporeal Life Support Organization Registry

**DOI:** 10.1186/s13054-022-04111-z

**Published:** 2022-08-03

**Authors:** Joseph E. Tonna, Chuen Seng Tan, Kasia Hryniewicz, Ryan P. Barbaro, Daniel Brodie, Graeme MacLaren

**Affiliations:** 1 The University of Utah School of Medicine, 30 N 1900 E, 3C127, Salt Lake City, UT 84132 USA; 2 National University of Singapore, Singapore, Singapore; 3 Minneapolis Heart Institute, Abbott Northwestern Hospital, Minneapolis, MN 84132 USA; 4 University of Michigan, Ann Arbor, MI 84132 USA; 5 Columbia University, New York, NY USA; 6 Cardiothoracic ICU, National University Hospital, Singapore, Singapore

**Keywords:** Cardiac failure, Respiratory failure, Acute respiratory distress syndrome, Myocardial inflammation

First described early in the pandemic, coronavirus disease 2019 (COVID-19)-associated fulminant myocarditis can present with arrhythmias and cardiogenic shock, but may be treatable with mechanical circulatory support, such as venoarterial (VA) extracorporeal life support (ECLS) [1]. To better characterize severe myocarditis during the COVID-19 pandemic, we analyzed adult patients with severe COVID-19 who received ECLS for cardiac, pulmonary, or combined failure using an international registry.

We calculated the rate of mechanical circulatory support use among patients receiving ECLS for COVID-19, describing patient characteristics and risk factors for mortality. We utilized the Extracorporeal Life Support Organization (ELSO) Registry and extracted data on patients ≥ 18 years old, diagnosed with COVID-19 from January 1, 2020, through March 31, 2021. Data released from ELSO are de-identified, do not meet the definition of Human Subjects Research, and therefore do not require repeat human subjects review.

Among 4792 patients receiving ECLS for COVID-19 (Table 1), 4.9% (234) were supported with VA-ECMO, and 88 patients (1.8%) had acute myocarditis during the study period. Among those with myocarditis, 35% were women (*p* = 0.092). COVID-19 patients receiving ECLS who were diagnosed with myocarditis (*n* = 88) were more likely to be managed with cardiac support versus pulmonary support (53% vs. 42%, *p* < 0.001). Among all patients supported for a cardiac indication, patients with myocarditis had less hypercarbia (PaCO_2_: 45 mmHg vs. 66 mmHg; *p* < 0.001), less hypoxemia (PaO_2_: 94 mmHg vs. 80 mmHg; *p* = 0.078), greater metabolic acidosis (serum bicarbonate 22 mEq/L vs. 28 mEq/L; *p* < 0.001), lower blood pressure (systolic: 101 mmHg vs. 119 mmHg; *p* < 0.001), and lower pulse pressure (43 mmHg vs. 55 mmHg; *p* < 0.001) compared with patients without myocarditis. Death percentages were similar between those with and without myocarditis.

**Table 1:** Differences in characteristics between COVID-19 patients on ECLS with and without myocarditis

Variable, *n* (%)		All^a^ (*n* = 4792)	Myocarditis^a^ (*n* = 88)	No myocarditis^a^ (*n* = 4704)	*P*-value
Age (years)^b^		49.2 (11.5)	47.6 (13.7)	49.2 (11.4)	0.187
Sex	Female	1305 (27.2)	31 (35.2)	1274 (27.1)	0.092
	Male	3487 (72.8)	57 (64.8)	3430 (72.9)	
Charlson comorbidity index (CCI)^b^		0.4 (0.8)	0.6 (0.8)	0.4 (0.8)	0.024
SAVE score^b^		− 6.7 (3.2)	− 5.2 (3.5)	− 6.7 (3.2)	0.001
Inotropes prior to ECLS	No	4643 (96.9)	62 (70.5)	4581 (97.4)	< 0.001
	Yes	149 (3.1)	26 (29.5)	123 (2.6)	
Vasopressors prior to ECLS	No	1975 (41.2)	15 (17)	1960 (41.7)	< 0.001
	Yes	2817 (58.8)	73 (83)	2744 (58.3)	
*Medical conditions*					
Asthma	No	4279 (89.3)	79 (89.8)	4200 (89.3)	1
	Yes	513 (10.7)	9 (10.2)	504 (10.7)	
Obesity (BMI > 30 kg/m^2^)	No	2309 (48.2)	57 (64.8)	2252 (47.9)	0.002
	Yes	2483 (51.8)	31 (35.2)	2452 (52.1)	
Diabetes	No	3349 (69.9)	67 (76.1)	3282 (69.8)	0.241
	Yes	1443 (30.1)	21 (23.9)	1422 (30.2)	
Chronic lung disease	No	4596 (95.9)	82 (93.2)	4514 (96)	0.174
	Yes	196 (4.1)	6 (6.8)	190 (4)	
Immunocompromised	No	4598 (96)	81 (92)	4517 (96)	0.09
	Yes	194 (4)	7 (8)	187 (4)	
Cancer	No	4705 (98.2)	84 (95.5)	4621 (98.2)	0.075
	Yes	87 (1.8)	4 (4.5)	83 (1.8)	
Chronic heart disease	No	4588 (95.7)	79 (89.8)	4509 (95.9)	0.012
	Yes	204 (4.3)	9 (10.2)	195 (4.1)	
Chronic renal insufficiency	No	4640 (96.8)	84 (95.5)	4556 (96.9)	0.362
	Yes	152 (3.2)	4 (4.5)	148 (3.1)	
Pregnancy	No	4683 (97.7)	87 (98.9)	4596 (97.7)	0.724
	Yes	109 (2.3)	1 (1.1)	108 (2.3)	
Frailty	No	4759 (99.3)	87 (98.9)	4672 (99.3)	0.459
	Yes	33 (0.7)	1 (1.1)	32 (0.7)	
*Physiologic values prior to ECLS*^*b*^					
C-reactive protein		124.4 (139)	107.2 (129.3)	124.7 (139.2)	0.414
pH		7.3 (0.1)	7.3 (0.1)	7.3 (0.1)	0.588
PaCO_2_ (mmHg)		65.5 (26.4)	45.2 (15.8)	65.9 (26.5)	< 0.001
PaO_2_ (mmHg)		79.9 (66.4)	94.1 (63.7)	79.7 (66.5)	0.078
Serum bicarbonate		28.3 (7.1)	21.5 (8.2)	28.4 (7)	< 0.001
Arterial saturation (%)		88.4 (9.6)	90.9 (9.1)	88.4 (9.6)	0.04
Systolic blood pressure (mmHg)		118.6 (24.7)	101 (28.7)	118.9 (24.5)	< 0.001
Diastolic blood pressure (mmHg)		63.7 (13.2)	58.4 (12.6)	63.7 (13.2)	0.001
Arterial pulse pressure (SBP-DBP)		54.9 (19.3)	42.5 (20.8)	55.2 (19.2)	< 0.001
*Administered medications*					
Systemic steroids	No	1398 (29.2)	31 (35.2)	1367 (29.1)	0.236
	Yes	3394 (70.8)	57 (64.8)	3337 (70.9)	
Remdesivir	No	2555 (53.3)	58 (65.9)	2497 (53.1)	0.018
	Yes	2237 (46.7)	30 (34.1)	2207 (46.9)	
Convalescent plasma	No	3362 (70.2)	74 (84.1)	3288 (69.9)	0.003
	Yes	1430 (29.8)	14 (15.9)	1416 (30.1)	
Chloroquine/hydroxychloroquine	No	4028 (84.1)	70 (79.5)	3958 (84.1)	0.24
	Yes	764 (15.9)	18 (20.5)	746 (15.9)	
Selective cytokine blockade (anakinra or tocilizumab)	No	3917 (81.7)	70 (79.5)	3847 (81.8)	0.578
	Yes	875 (18.3)	18 (20.5)	857 (18.2)	
JAK inhibition	No	4769 (99.5)	87 (98.9)	4682 (99.5)	0.348
	Yes	23 (0.5)	1 (1.1)	22 (0.5)	
Intravenous immunoglobulin	No	4708 (98.2)	81 (92)	4627 (98.4)	0.001
	Yes	84 (1.8)	7 (8)	77 (1.6)	
*ECLS support type*					
Cardiac		175 (3.7)	47 (53.4)	128 (2.7)	< 0.001
ECPR		51 (1.1)	4 (4.5)	47 (1)	
Pulmonary		4566 (95.3)	37 (42)	4529 (96.3)	
*First ECLS support type*					
Other		10 (0.2)	1 (1.1)	9 (0.2)	< 0.001
VenoArterial		234 (4.9)	46 (52.3)	188 (4)	
VenoPulmonary		44 (0.9)	0 (0)	44 (0.9)	
VenoVeno		4466 (93.2)	32 (36.4)	4434 (94.3)	
VenoVenoArterial		38 (0.8)	9 (10.2)	29 (0.6)	
Survived	No	2551 (53.2)	45 (51.1)	2506 (53.3)	0.747
	Yes	2241 (46.8)	43 (48.9)	2198 (46.7)	

*BMI* body mass index, *CCI* Charlson comorbidity index, *COVID-19* coronavirus disease 2019, *DBP* diastolic blood pressure, *ECPR* extracorporeal cardiopulmonary resuscitation, *ECLS* extracorporeal life support, *JAK* Janus kinase, *PaCO*_*2*_ partial pressure of arterial carbon dioxide, *PaO*_*2*_ partial pressure of arterial oxygen, *SAVE* survival after venoarterial ECMO, *SBP* systolic blood pressure

^a^column percent for variables with *n*, %

^b^value, (standard deviation [SD])

We acknowledge limitations to this analysis. The Registry did not require myocardial biopsy to prove myocarditis, and hence, patients may have had myocarditis diagnosed as acutely decreased myocardial function on echocardiography accompanied by elevated cardiac enzymes and/or electrocardiographic changes. To account for potential overdiagnosis of myocarditis, we performed a sensitivity analysis excluding patients coded for acute myocardial infarction (AMI). The results were unchanged.

In summary, among patients with COVID-19 supported with ECLS, a diagnosis of myocarditis was uncommon (1.8%) and mortality was 51%. In context, mortality among ECLS patients in the ELSO Registry for COVID-19 was 37% early in the pandemic, and 52% later [2]. This is compared to 25–42% mortality for critically ill COVID-19 patients without ECLS [3]. Risk factors for death among patients with myocarditis receiving ECLS were increasing age and preexisting diabetes mellitus. These findings are consistent with previously identified mortality risk factors in non-ECLS patients with severe COVID-19 [3].

Our findings are important for a number of reasons. While within many high-income nations, high rates of vaccination have greatly reduced mortality, across the world vaccination rates remain only 60% and the pandemic is not over. Our results are the largest COVID-19 myocarditis case series using ECLS and may inform future outbreaks. Finally, we identified that risk factors for mortality among patients with myocarditis on ECLS are not different than among patients without myocarditis. In conclusion, within the largest international Registry of patients requiring ECLS circulatory support for COVID-19, mortality appears higher than for patients with COVID-19 without ECLS, but no different than those on ECLS with only acute respiratory failure.

## Data Availability

Data were analyzed under contract from ELSO and can be requested from ELSO directly.

## Abbreviations

COVID-19: Coronavirus disease 2019
ECLS: Extracorporeal life support
ELSO: Extracorporeal Life Support Organization
AMI: Acute myocardial infarction
